# Artificial Intelligence and Advanced Digital Health for Hypertension: Evolving Tools for Precision Cardiovascular Care

**DOI:** 10.3390/medicina61091597

**Published:** 2025-09-04

**Authors:** Ioannis Skalidis, Niccolo Maurizi, Adil Salihu, Stephane Fournier, Stephane Cook, Juan F. Iglesias, Pietro Laforgia, Livio D’Angelo, Philippe Garot, Thomas Hovasse, Antoinette Neylon, Thierry Unterseeh, Stephane Champagne, Nicolas Amabile, Neila Sayah, Francesca Sanguineti, Mariama Akodad, Henri Lu, Panagiotis Antiochos

**Affiliations:** 1 Institut Cardiovasculaire Paris-Sud, Hôpital Jacques Cartier, Ramsay-Santé, 91300 Massy, France; 2 School of Medicine, University of Crete, 71500 Heraklion, Greece; 3 Service of Cardiology, Lausanne University Hospital and University of Lausanne, 1005 Lausanne, Switzerlandstephane.fournier@chuv.ch (S.F.);; 4 Cardiology Department, University and Hospital Fribourg, 1700 Fribourg, Switzerland; 5 Division of Cardiology, University Hospital Geneva, 1211 Geneva, Switzerland; juanfernando.iglesias@hug.ch

**Keywords:** hypertension, artificial intelligence, digital health, remote monitoring, digital twins, precision medicine

## Abstract

*Background*: Hypertension remains the leading global risk factor for cardiovascular morbidity and mortality, with suboptimal control rates despite guideline-directed therapies. Digital health and artificial intelligence (AI) technologies offer novel approaches for improving diagnosis, monitoring, and individualized treatment of hypertension. *Objectives*: To critically review the current landscape of AI-enabled digital tools for hypertension management, including emerging applications, implementation challenges, and future directions. *Methods*: A narrative review of recent PubMed-indexed studies (2019–2024) was conducted, focusing on clinical applications of AI and digital health technologies in hypertension. Emphasis was placed on real-world deployment, algorithmic explainability, digital biomarkers, and ethical/regulatory frameworks. Priority was given to high-quality randomized trials, systematic reviews, and expert consensus statements. *Results*: AI-supported platforms—including remote blood pressure monitoring, machine learning titration algorithms, and digital twins—have demonstrated early promise in improving hypertension control. Explainable AI (XAI) is critical for clinician trust and integration into decision-making. Equity-focused design and regulatory oversight are essential to prevent exacerbation of health disparities. Emerging implementation strategies, such as federated learning and co-design frameworks, may enhance scalability and generalizability across diverse care settings. *Conclusions*: AI-guided titration and digital twin approaches appear most promising for reducing therapeutic inertia, whereas cuffless blood pressure monitoring remains the least mature. Future work should prioritize pragmatic trials with equity and cost-effectiveness endpoints, supported by safeguards against bias, accountability gaps, and privacy risks.

## 1. Introduction

Hypertension remains the leading modifiable risk factor for cardiovascular disease (CVD), responsible for over 10 million deaths globally each year [1]. Despite the availability of effective pharmacologic therapies and evidence-based guidelines, real-world blood pressure (BP) control rates continue to stagnate, with fewer than 25% of hypertensive adults achieving target values in many regions [2]. This persistent treatment gap reflects multifactorial barriers, including poor medication adherence, therapeutic inertia, fragmented care delivery, and limited health system capacity [3,4].

In response to these challenges, digital health technologies have emerged as scalable, patient-centered tools capable of augmenting conventional hypertension care [5]. First-generation solutions—such as mobile health (mHealth) applications, telemonitoring platforms, SMS reminders, and wearable devices—have demonstrated modest but consistent efficacy in improving BP control and treatment adherence, particularly when integrated into primary care workflows [6,7]. However, these interventions are often limited by their reactive nature, low personalization, and insufficient integration into therapeutic decision-making.

Concurrently, the rapid evolution of artificial intelligence (AI) and related computational sciences is opening new frontiers in the management of chronic diseases. In hypertension, AI-driven applications have begun to transition from prediction models and risk stratification to real-time decision support, medication self-titration algorithms, and virtual physiological models. These advances enable more dynamic and individualized treatment paradigms, shifting from episodic clinic-based care to continuous, data-driven disease management [8,9].

Among the most transformative concepts is the development of digital twins—computational replicas of individual patients that integrate biometric, behavioral, and clinical data to simulate disease progression and treatment response [10]. Although still in early phases of application in hypertension, digital twin frameworks are being explored for therapy optimization, remote titration protocols, and clinical trial simulation [11].

Although recent narrative reviews have explored the role of artificial intelligence in cardiovascular medicine more broadly, none has provided a comprehensive synthesis focused specifically on hypertension. Existing reviews often emphasize applications in arrhythmia detection or cardiovascular imaging, but they do not systematically address the unique challenges of hypertension management, such as therapeutic inertia, remote monitoring, and the integration of digital twins or large language models. This review therefore fills an important gap by bringing together these emerging domains in the context of guideline-based hypertension care.

**Figure 1:**
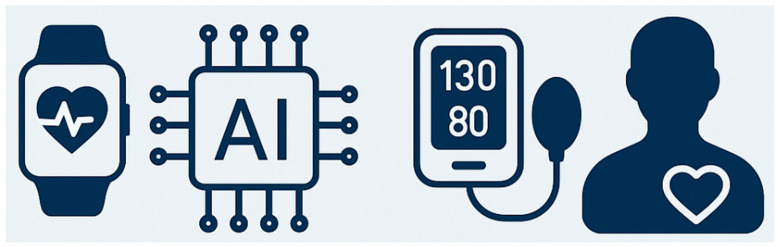
**Artificial intelligence and digital technologies in cardiovascular care**. Conceptual illustration of AI-integrated cardiovascular care, including wearable cardiac monitoring, algorithmic data processing, automated blood pressure measurement, and individualized patient management. AI—artificial intelligence.

The objective of this review is to critically evaluate current and emerging applications of artificial intelligence and digital health technologies in hypertension, with a focus on remote monitoring, medication titration, digital biomarkers, and simulation-based approaches. We aim to highlight the evidence base, identify methodological and ethical challenges, and outline future research directions needed to translate these tools into routine clinical practice (Figure 1).

## 2. Methods

This work is a narrative review conducted in accordance with best practices for non-systematic evidence syntheses, with explicit description of search strategy, selection criteria, and synthesis approach to enhance transparency.

### 2.1. Databases and Search Strategy

A structured literature search was performed in PubMed/MEDLINE for studies published between 1 January 2019 and 31 December 2024. The following Boolean search string was used: (“hypertension” OR “arterial hypertension” OR “high blood pressure”) AND (“artificial intelligence” OR “machine learning” OR “deep learning” OR “large language model” OR “digital health” OR “digital biomarker” OR “remote monitoring” OR “digital twin” OR “wearable blood pressure”). Search terms were adapted from Medical Subject Headings (MeSH) and free-text keywords. Additional relevant articles were identified through citation tracking and from the authors’ personal libraries.

### 2.2. Eligibility Criteria

Eligible studies included peer-reviewed publications on adults with hypertension or elevated blood pressure that evaluated AI-enabled or digital health tools with clinical relevance (e.g., decision support, medication titration algorithms, cuffless BP monitoring, digital twins, LLM-based adherence support). We considered randomized controlled trials, cohort studies, systematic reviews, meta-analyses, pilot studies, and expert consensus statements. Non–peer-reviewed material, commentaries without data, and conference abstracts were excluded.

### 2.3. Synthesis Approach

Data from included studies were synthesized narratively into four domains: (1) AI-guided blood pressure titration, (2) digital twins and patient-specific modeling, (3) digital biomarkers and remote monitoring, and (4) ethical, regulatory, and equity considerations. Within each domain, methodological robustness, generalizability, and readiness for routine clinical use were critically appraised.

## 3. AI-Guided Remote Blood Pressure Titration

**Figure 2:**
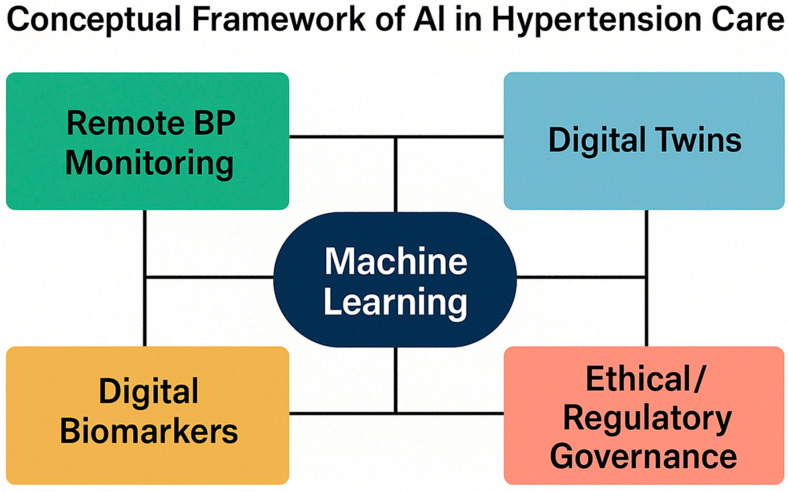
**Conceptual framework of AI in hypertension care, including monitoring, titration, and strategies to overcome therapeutic inertia**. Core domains of AI integration in hypertension management, including machine learning, remote BP monitoring, digital biomarkers, digital twins, and ethical/regulatory governance. AI—artificial intelligence; BP—blood pressure.

Timely therapeutic adjustment remains one of the most significant barriers to effective blood pressure control in routine clinical practice, a challenge that AI-enabled frameworks aim to address through improved monitoring and titration (Figure 2). Despite the availability of evidence-based antihypertensive therapies, many patients experience prolonged periods of undertreatment due to clinical inertia, infrequent follow-up, and healthcare resource constraints. In recent years, digital health technologies have emerged as promising tools to close this gap. Among these, AI-based systems for remote medication titration represent a novel frontier in precision hypertension care.

The concept of self-titration supported by home blood pressure monitoring was first validated in the TASMIN-SR randomized controlled trial, which demonstrated that patients using structured self-monitoring protocols achieved significantly greater reductions in systolic blood pressure compared to those receiving usual care (mean difference: −5.4 mmHg at 12 months) [12]. While TASMIN-SR did not employ AI algorithms, it established a foundational model for safe and effective medication adjustment outside traditional clinical settings.

Building on this framework, AI-assisted titration platforms are now being developed to personalize antihypertensive therapy by adapting to the dynamic clinical profile of each patient. These models are designed to analyze longitudinal data—including daily blood pressure readings, prior drug responses, and patient-specific characteristics—to generate dose recommendations that evolve in real time. One of the first AI systems formally tested in this context is CURATE.AI, which constructs individualized dose–response maps using a small number of response-outcome data points. This mechanism-independent approach allows for dynamic, fine-tuned titration while minimizing pharmacologic burden.

The feasibility of CURATE.AI for antihypertensive therapy was formally evaluated in the CURATE.AI ADAPT pilot trial, a multi-arm, randomized protocol published in 2023 [13]. Although full outcome data are pending, the study represents the first peer-reviewed, clinical application of an AI-based dose titration system in hypertension. Its results are expected to inform future development of adaptive platforms that balance personalization with safety and potentially reduce the need for frequent clinician intervention.

To date, no other AI platforms for autonomous or semi-autonomous blood pressure titration have been validated in randomized trials or published in indexed journals. Several investigational systems are under development, and ongoing trials such as AI-SMART are anticipated to expand the evidence base. However, these should be interpreted cautiously until peer-reviewed results become available.

The 2024 ESC/ESH Guidelines for the Management of Arterial Hypertension endorse structured home monitoring combined with therapeutic adjustment under clinical supervision as a Class IIa, Level B recommendation [14]. This further supports the integration of remote titration strategies into contemporary hypertension management. AI systems designed for this purpose may ultimately offer scalable, personalized solutions that complement traditional care pathways, especially in primary care and underserved settings.

To ensure safety and consistency, AI titration logic should explicitly mirror the 2024 ESC/ESH stepwise algorithm: (i) confirm diagnosis and out-of-office BP, (ii) initiate first-line combinations in most patients, (iii) intensify to triple therapy when targets are not achieved, and (iv) escalate to resistant-hypertension pathways as indicated. Decision engines can operationalize these steps by triggering dose escalation or combination therapy when average home or ambulatory BP remains above individualized targets over a predefined interval, while simultaneously checking comorbidity-specific preferences (e.g., CKD, HF, CAD), drug–drug interactions, and prior response patterns. Such designs preserve guideline fidelity yet personalize choices for multimorbidity and polypharmacy, with clinician oversight retained for exceptions and safety checks [14,15].

Nevertheless, the clinical adoption of AI-based titration remains in its infancy. Key challenges include ensuring model generalizability across diverse populations, integrating AI systems with electronic health records, and addressing concerns regarding algorithm transparency and clinical accountability [16]. As these technologies advance, the emphasis must shift toward regulatory evaluation, clinician oversight frameworks, and prospective studies demonstrating long-term impact on cardiovascular outcomes (Table 1).

## 4. Digital Twin Technologies in Hypertension: Concept and Applications

Digital twin technology in medicine refers to a virtual, continuously updated model of an individual patient, constructed to simulate physiological responses and disease progression under various interventions. While most existing digital twin implementations in cardiology have focused on electrophysiology or structural heart disease, their underlying frameworks are directly applicable to hypertension care. Notably, digital twins have already been deployed at scale in cardiovascular imaging and disease modeling, offering a pathway toward precision management of elevated blood pressure [17].

A detailed mapping review analyzed 88 digital twin applications across cardiovascular domains, highlighting their emergent role in personalized risk prediction, digital phenotyping, and virtual trial simulations—though only a minority addressed blood pressure or hypertension specifically [18]. Complementing this, another review in 2024 provided foundational theory, describing how hybrid mechanistic-statistical modeling approaches can integrate patient data—from imaging, physiology, and wearables—to enable individualized simulation and decision support [19].

Conceptually, constructing a hypertension-specific digital twin requires longitudinal integration of demographics, comorbidities, medication history, and ambulatory or home BP data. Enhanced fidelity may be achieved via inclusion of wearable-derived physiologic signals (e.g., vascular stiffness indices), behavioral data, and pharmacogenomics. Similar hybrid mechanistic–statistical approaches have already been described in cardiovascular modeling [10,18]. Through computational frameworks such as inverse mechanistic modeling or reinforcement learning, treatment trajectories can be simulated in silico, enabling clinicians to compare scenarios prior to initiation [19]. Yet, significant implementation challenges persist, including interoperability gaps, limited diversity in training datasets, and regulatory uncertainty. Ethical considerations—including privacy, bias, and liability—also require proactive governance before deployment.

In summary, while digital twin–driven management of hypertension has not yet been validated in dedicated RCTs, the emerging architecture and real-world outcomes from related digital twin interventions provide a credible roadmap [20]. Future research should prioritize RCTs targeting hypertension cohorts, integration with AI-guided titration systems, and validation across varied demographic settings. Hybrid platforms combining predictive simulation, adaptive dosing, and behavioral personalization may represent the transitional model toward fully integrated digital twin hypertension care.

## 5. Digital Biomarkers and Remote Blood Pressure Monitoring in Hypertension

Advances in wearable and ECG technology have created objective digital biomarkers that capture blood pressure–related physiology outside of the clinic. These tools offer objective insights into hypertension and may support early detection, risk stratification, and treatment monitoring.

A systematic review and meta-analysis evaluated six randomized controlled trials involving cuffless wearable blood pressure devices. The pooled standardized mean differences were small and not statistically significant, highlighting device variability, validation concerns, and inconsistent adherence [21]. Nevertheless, these wearable systems represent a significant step toward continuous, non-invasive blood pressure assessment.

Next-generation ECG-based AI models such as HTN-AI have demonstrated that deep learning applied to 12-lead ECGs can reliably discriminate individuals with hypertension and predict future cardiovascular events. In internal and external validations, HTN-AI achieved AUCs of 0.803 and 0.771, respectively, and predicted risk of heart failure, myocardial infarction, stroke, and mortality with hazard ratios up to 2.26 per standard deviation increase [22]. HTN-AI thus serves as a promising digital biomarker that captures subclinical physiological effects of elevated blood pressure.

Beyond these examples, the field remains nascent. Consensus definitions emphasize that digital biomarkers must be objectively measured, algorithmically processed, and linked to meaningful clinical outcomes [23]. In hypertension—in digital therapeutics and remote interventions—recent randomized trials such as HERB-DH1 and the HALCYON RCT demonstrate promise for BP self-management apps in lowering systolic pressure [24,25]. Yet challenges persist—telehealth systems must ensure device accuracy, integrate seamlessly with electronic health records, and remain accessible across diverse populations; robust validation and equitable implementation are crucial.

Except for accuracy, prospective validation and real-world cohort studies paint a cautious picture. In a head-to-head 24 h study of a popular consumer-grade cuffless wearable (Aktiia) versus a medical-grade ambulatory BP monitor (Mobil-O-Graph) in 31 adults, agreement was poor to moderate (Bland–Altman limits ≈ −30/+30 mmHg for SBP and −20/+15 mmHg for DBP), nocturnal dipping and BP variability were underestimated, and high-BP detection achieved ~50% sensitivity and ~80% specificity; the authors concluded the cuffless device should not replace standard 24 h ABPM at present [26]. Complementing this, a pragmatic clinical practice cohort evaluating a pulse-transit-time cuffless device (Somnotouch-NIBP) showed limited ability to track BP changes after calibration—the larger the true BP change, the larger the divergence from cuff-based ABPM [27]. These findings align with the European Society of Hypertension (ESH) recommendations, which require multi-condition validation (static accuracy, body position, treatment-induced BP change, awake/asleep change, exercise response, and re-calibration stability) before any cuffless device is considered for clinical use [28]. Taken together, cuffless monitoring may be useful for dense phenotyping and engagement as an adjunct within supervised digital pathways, but robust, outcome-focused pragmatic randomized trials are still needed before routine replacement of ABPM/HBPM. Thus, while promising for research and engagement, cuffless monitoring remains insufficiently validated for clinical endpoints and should be viewed as exploratory.

## 6. Clinical Applications and Outcomes of AI-Driven Hypertension Management

The clinical translation of artificial intelligence in hypertension management has primarily materialized through decision support systems, digital coaching programs, and early-stage adaptive algorithms for blood pressure titration. While fully autonomous, AI-based pharmacological treatment systems remain in development, several pragmatic trials demonstrate how algorithm-guided interventions can improve therapeutic alignment, patient engagement, and BP control.

Computerized clinical decision support systems (CDSS) represent the earliest form of AI integration in routine care. In a large cluster-randomized trial involving 2111 hypertensive patients across 14 practices, Hicks et al. demonstrated that a CDSS intervention significantly improved adherence to guideline-directed antihypertensive prescribing (7% vs. 5%; OR 1.39, *p* < 0.001) compared to usual care, though no significant differences were observed in absolute BP control after 18 months of follow-up [29]. These findings highlight the limitations of provider-facing algorithmic tools when not paired with patient-level behavioral or monitoring components.

More recent developments have focused on autonomous digital interventions combining remote monitoring with adaptive feedback. In a non-randomized real-world study of 141 adults with uncontrolled hypertension, a fully digital, AI-driven lifestyle coaching program—integrating wearable BP monitors, behavioral prompts, and tailored messaging—achieved mean reductions of 5.6 mmHg in systolic and 3.8 mmHg in diastolic BP at 12 weeks [30]. Among participants with stage 2 hypertension, reductions reached 14.2 mmHg systolic and 8.1 mmHg diastolic by 24 weeks. Importantly, >90% of users remained adherent, and clinician intervention was limited to <6% of participants, underscoring the potential for scalability in resource-limited settings.

A growing body of research has evaluated large language models (LLMs) for drafting patient messages and supporting triage in chronic care settings. In prospective quality-improvement studies, EHR-integrated LLMs were associated with reduced clinician task load and longer, more personalized replies, though oversight remained essential [31]. Comparative evaluations in emergency medicine have shown triage proficiency comparable to untrained physicians, with pooled meta-analytic accuracy around 0.86 (95% CI 0.64–0.98) [32]. While these investigations were not hypertension-specific, they illustrate how conversational AI could be adapted to remote hypertension programs to triage abnormal blood pressure uploads, reinforce adherence messaging, and sustain patient engagement between clinical encounters. Dedicated hypertension-focused evaluations of LLM platforms are still lacking and represent an important area for future research.

A review synthesizing AI applications across the hypertension care continuum—including algorithmic screening, risk stratification, cuffless BP estimation, and personalized behavioral support. The review underscores that while AI-enabled platforms consistently improve process measures such as prescribing and engagement, their direct effect on hard outcomes (e.g., cardiovascular events, medication intensification) remains understudied in large-scale randomized settings [33].

Notably, while early-phase platforms show promise, limitations persist. Many interventions rely on proprietary algorithms without external validation, and few incorporate explainability frameworks to support clinician trust. Regulatory guidance remains fragmented, and health equity considerations—particularly around access to connected devices—are insufficiently addressed in current deployment strategies [34].

Future directions must prioritize prospective randomized trials of AI-supported treatment platforms that combine dynamic BP tracking with pharmacologic titration, ideally embedded within electronic health records and supported by interoperable infrastructures. These tools should be rigorously tested not only for BP reduction, but also for their impact on adherence, treatment intensification, cardiovascular event reduction, and cost-effectiveness (Table 2).

## 7. Implementation, Ethical Governance, and Future Directions in AI-Driven Hypertension Management

Despite promising innovations, the real-world integration of AI-enabled digital tools for hypertension care is hindered by intertwined technical, ethical, and health system challenges. Key barriers—limited interoperability, inadequate clinician training, user resistance, and increased workload—were identified with relative frequency of occurrence ranging from 3.9% to 6.4% across systematic reviews [33]. These findings underscore the need for infrastructure readiness, clinician-centered design, and streamlined workflow integration to support adoption.

Health equity remains a vital consideration. A meta-analysis confirmed that culturally tailored digital hypertension interventions yielded significant systolic blood pressure reductions (~4 mmHg at six months) in underserved U.S. populations [34]. These results emphasize that equity-focused design, including collaboration with community health workers and language-appropriate interfaces, is essential to prevent technology-driven disparities.

Ethical governance is essential for the safe deployment of AI in hypertension care. Explainable AI (XAI) has emerged as a foundational strategy to increase the interpretability of predictive algorithms, promote clinician trust, and reduce risks associated with opaque “black box” systems [35]. Formalized frameworks for XAI allow clinicians to inspect, override, and validate AI insights, which is especially critical when AI supports pharmacologic titration decisions.

Beyond interpretability, several specific ethical dilemmas are particularly relevant to hypertension AI. Most titration algorithms are trained on relatively homogeneous cohorts, raising the risk of algorithmic bias and reduced accuracy in elderly patients, ethnic minorities, or those with multimorbidity [36]. Questions of accountability also remain unresolved: if an AI-driven titration suggestion contributes to harm, it is unclear whether responsibility lies with the clinician, the developer, or the health system. Continuous blood pressure monitoring and wearable-derived biomarkers further introduce novel privacy and data security risks, particularly when data are shared across commercial platforms [37]. Addressing these challenges will require transparent reporting, ongoing performance audits, and governance structures that clearly define liability and ensure equitable benefit.

Regulatory evolution has begun to address AI’s unique challenges. The U.S. FDA’s proposed framework for Software as a Medical Device (SaMD) introduces a “predetermined change control plan” to govern algorithm updates and post-market performance surveillance [38]. Parallel initiatives in the European Union via the Artificial Intelligence Act propose risk-tiered regulation of clinical decision support systems, emphasizing transparency, external auditing, and documentation. Although these frameworks are not yet hypertension-specific, they set the stage for safe deployment of AI in clinical care.

Experience from coronary artery disease, heart failure, and arrhythmia care shows that successful AI translation depends on clinician co-design, prospective validation, and interpretability. For example, AI-enabled ECG algorithms have been prospectively validated for detecting left ventricular dysfunction [39], while recent review stress continuous performance monitoring and transparency as prerequisites for deployment [40]. The same principles apply to hypertension: embedding interpretability, rigorous real-world testing, and workflow co-design will be essential for AI titration and remote monitoring. Comparable lessons have been highlighted in a recent commentary on AI in cardiology, which underscored the value of cross-domain learning [41]. However, most available studies remain small or non-randomized, and evidence for hard outcomes such as cardiovascular events is still lacking.

Implementation must also consider system-level factors for scalability. Federated learning approaches have demonstrated value in multi-center cardiovascular studies, enabling collaborative model training without central data sharing—a privacy-preserving architecture that supports generalizability across health systems [42]. Similarly, clinical decision-support interventions require alignment with reimbursement pathways and health policy; pragmatic trials suggest that interventions tied to digital health coverage can significantly increase provider adherence to guidelines [43].

Economic evidence for AI-enabled hypertension care remains limited but informative. A 2019 model-based evaluation accompanying the TASMINH4 trial suggested that physician titration guided by self-monitored blood pressure—particularly when paired with telemonitoring—was likely cost-effective over usual care at commonly accepted UK thresholds, although uncertainty persisted around long-term assumptions [44]. More recently, a 2023 systematic review of 16 studies concluded that home blood pressure monitoring (HBPM) is generally more cost-effective than clinic-based monitoring over ≥10-year horizons, with the greatest value seen when HBPM is combined with additional team-based support; however, heterogeneity in methods and perspectives was substantial [45]. Emerging digital therapeutics may also be cost-effective; a lifetime analysis using HERB-DH1 randomized trial inputs estimated an incremental cost-effectiveness ratio of approximately ¥1.2 million (~USD 10,400) per QALY for app-based hypertension management versus lifestyle counseling alone, with results sensitive to attrition and subscription price [46]. Collectively, these data support incorporating prospective cost-utility and budget-impact endpoints into future pragmatic trials of AI-guided titration and remote monitoring to inform payer decisions and reimbursement pathways.

Looking forward, successful deployment of AI-driven hypertension platforms will require prospective multicenter trials that integrate biometric monitoring, explainable algorithmic support, and supervised medication titration. The inclusion of federated learning strategies may support broader model applicability while preserving patient data privacy. Co-design partnerships with clinicians, patients, technologists, and ethicists will safeguard relevance, trust, and usability [42]. Equity-focused planning—attending to access, literacy, and language diversity—is essential to ensure inclusive benefit. Ultimately, alignment with evolving regulatory frameworks and reimbursement models will determine sustainability and impact at scale (Table 3 and Table 4).

## 8. Limitations and Strengths

This narrative review provides a contemporary synthesis of AI-enabled digital health applications in hypertension, with emphasis on clinical integration and precision care pathways. Key strengths include the use of a structured search strategy, thematic synthesis of diverse technologies, and critical appraisal of both established and emerging tools.

However, several limitations should be acknowledged. First, the search was limited to PubMed/MEDLINE and English-language publications, introducing potential selection bias. Second, as a narrative review, the methodology does not incorporate systematic quality scoring or quantitative synthesis, which may limit the precision of effect estimates. Third, the evidence base for some technologies—such as digital twins and cuffless blood pressure devices—remains preliminary, and findings from early-phase or pilot studies may not generalize to broader populations. Finally, rapid technological evolution means that new developments may have emerged since the time of the literature search.

These limitations underscore the need for ongoing, methodologically rigorous reviews and meta-analyses as the field matures.

## 9. Conclusions

Artificial intelligence and digital health are beginning to transform hypertension management, but their maturity varies. The most promising applications are AI-guided titration platforms aligned with ESC/ESH guidelines and emerging digital twin models, which directly address therapeutic inertia and support personalized care. By contrast, cuffless blood pressure monitoring remains the least mature field, with persistent concerns about accuracy and validation. Future progress requires pragmatic multicenter randomized trials in diverse populations, including cost-effectiveness and equity endpoints. Importantly, development and deployment must be coupled with safeguards addressing algorithmic bias, accountability, and privacy to ensure that these technologies deliver safe, equitable, and sustainable improvements in hypertension care.

## Figures and Tables

**Table 1:** **Overview of AI-enabled digital health interventions for hypertension management.** This table summarizes the core functionalities, applications, clinical impact, and supporting evidence of various AI and digital technologies currently explored for hypertension care. AI, Artificial Intelligence; BP, Blood Pressure; ML, Machine Learning; XAI, Explainable Artificial Intelligence; RCT, Randomized Controlled Trial.

Technology	Function	Example Applications	Clinical Outcomes	Level of Evidence
**Remote BP Monitoring**	Continuous or home-based BP tracking	Smartphone apps with Bluetooth cuffs	Improved BP control, enhanced adherence	RCTs, meta-analyses
**Machine Learning Algorithms**	Risk prediction, medication titration	ML models predicting uncontrolled BP or optimizing drug regimens	Increased treatment intensification, reduced variability	Observational + pilot RCTs
**Digital Twins**	Personalized simulations of BP response	Virtual BP phenotype to simulate therapy effects	Theoretical benefit; limited clinical validation	Conceptual/early clinical
**Explainable AI (XAI)**	Transparent decision support	Visual explanations for BP prediction models	Enhanced clinician trust, interpretability	Preclinical and pilot trials
**Federated Learning**	Multi-center model training without data sharing	Cross-hospital AI models for BP titration	Maintains privacy, supports scalability	Early implementation studies

**Table 2:** **Summary of Clinical Trials and Meta-Analyses Evaluating Digital Interventions in Hypertension.** Overview of selected high-quality clinical trials and meta-analyses evaluating the effectiveness of digital health interventions—including mobile applications, remote monitoring, and telemedicine platforms—on blood pressure control outcomes. BP, Blood Pressure; CHW, Community Health Worker; CQO, Cardiovascular Quality and Outcomes; JMIR, Journal of Medical Internet Research; SMS, Short Message Service.

Study (First Author, Year)	Intervention	Comparator	Population	BP Reduction (mmHg)	Duration	Key Findings
**Chow et al., 2022 [4]**	SMS-based reminders	Control	Latin America	−2.2 systolic	1 year	Modest benefit
**Omboni et al., 2020 [5]**	Team-based digital titration	Office BP follow-up	US primary care	−7.0 systolic	6 months	Increased treatment intensification
**Morawski et al., 2018 [8]**	Smartphone BP app	Standard care	Chinese adults with hypertension	−5.3 systolic	12 months	Enhanced self-management
**Katz et al., 2024 [34]**	Tailored mHealth + CHW	Usual care	Underserved US adults	−4.1 systolic	6 months	Improved equity and control

**Table 3:** **Key Barriers and Enablers for AI-Based Hypertension Management.** Key systemic, technological, and regulatory barriers to implementation of AI-driven hypertension management platforms, along with proposed enablers and supporting literature. AI, Artificial Intelligence; CHW, Community Health Worker; HER, Electronic Health Record; ESH, European Society of Hypertension; EU, European Union; FDA, U.S. Food and Drug Administration; SaMD, Software as a Medical Device.

Domain	Barrier	Enabler	Source
**Infrastructure**	Lack of EHR integration	Interoperable platforms	Nascimento et al., NPJ Digit Med 2023 [33]
**Clinician Engagement**	Low trust in black-box models	Explainable AI frameworks	Sadeghi et al., Comput Biol Med 2024 [35]
**Equity**	Digital literacy gaps, device access	Tailored interfaces, CHW support	Katz et al., JAMA Netw Open 2024 [34]
**Regulation**	Dynamic algorithms hard to audit	FDA SaMD framework, EU AI Act	US FDA 2019; Stergiou et al., J Hypertens 2023 [28,38]
**Data Privacy**	Sharing concerns across institutions	Federated learning architecture	Nascimento et al., NPJ Digit Med 2023 [33]

**Table 4:** **Future Research Priorities in Digital Hypertension Management.** Proposed directions to guide future research and implementation of AI-supported digital platforms for hypertension, emphasizing clinical validation, scalability, and equity. AI, Artificial Intelligence; BP, Blood Pressure; RCT, Randomized Controlled Trial.

Priority Area	Proposed Direction	Rationale
**Prospective RCTs**	Evaluate AI + remote BP tools in diverse settings	Clinical validation, generalizability
**Health Economic Studies**	Assess cost-effectiveness of digital hypertension tools	Reimbursement, policy alignment
**Implementation Science**	Study uptake in low-resource settings	Address global disparities
**Co-design Strategies**	Involve clinicians, patients in model development	Improve trust, usability

## Data Availability

Data available upon request to the corresponding author.

## Abbreviations

AI	Artificial Intelligence
BP	Blood Pressure
CDSS	Clinical Decision Support System
HBPM	Home Blood Pressure Monitoring
LLM	Large Language Model
RCT	Randomized Controlled Trial
